# Molecular Determinants for Photodynamic Therapy Resistance and Improved Photosensitizer Delivery in Glioma

**DOI:** 10.3390/ijms25168708

**Published:** 2024-08-09

**Authors:** David Aebisher, Paweł Woźnicki, Magdalena Czarnecka-Czapczyńska, Klaudia Dynarowicz, Ewelina Szliszka, Aleksandra Kawczyk-Krupka, Dorota Bartusik-Aebisher

**Affiliations:** 1 Department of Photomedicine and Physical Chemistry, Medical College of The Rzeszów University, 35-310 Rzeszów, Poland; 2 English Division Science Club, Medical College of The Rzeszów University, 35-310 Rzeszów, Poland; pw118616@stud.ur.edu.pl; 3 Department of Internal Medicine, Angiology and Physical Medicine, Center for Laser Diagnostics and Therapy, Medical University of Silesia, Batorego 15 Street, 41-902 Bytom, Poland; magdalena.czarnecka921114@gmail.com; 4 Center for Innovative Research in Medical and Natural Sciences, Medical College of The University of Rzeszów, 35-310 Rzeszów, Poland; kdynarowicz@ur.edu.pl; 5 Department of Microbiology and Immunology, Medical University of Silesia, Poniatowskiego 15, 40-055 Katowice, Poland; eszliszka@sum.edu.pl; 6 Department of Biochemistry and General Chemistry, Medical College of The Rzeszów University, 35-310 Rzeszów, Poland; dbartusik-aebisher@ur.edu.pl

**Keywords:** photodynamic therapy (PDT), glioma, molecular insights, treatment cancer, oncology

## Abstract

Gliomas account for 24% of all the primary brain and Central Nervous System (CNS) tumors. These tumors are diverse in cellular origin, genetic profile, and morphology but collectively have one of the most dismal prognoses of all cancers. Work is constantly underway to discover a new effective form of glioma therapy. Photodynamic therapy (PDT) may be one of them. It involves the local or systemic application of a photosensitive compound—a photosensitizer (PS)—which accumulates in the affected tissues. Photosensitizer molecules absorb light of the appropriate wavelength, initiating the activation processes leading to the formation of reactive oxygen species and the selective destruction of inappropriate cells. Research focusing on the effective use of PDT in glioma therapy is already underway with promising results. In our work, we provide detailed insights into the molecular changes in glioma after photodynamic therapy. We describe a number of molecules that may contribute to the resistance of glioma cells to PDT, such as the adenosine triphosphate (ATP)-binding cassette efflux transporter G2, glutathione, ferrochelatase, heme oxygenase, and hypoxia-inducible factor 1. We identify molecular targets that can be used to improve the photosensitizer delivery to glioma cells, such as the epithelial growth factor receptor, neuropilin-1, low-density lipoprotein receptor, and neuropeptide Y receptors. We note that PDT can increase the expression of some molecules that reduce the effectiveness of therapy, such as Vascular endothelial growth factor (VEGF), glutamate, and nitric oxide. However, the scientific literature lacks clear data on the effects of PDT on many of the molecules described, and the available reports are often contradictory. In our work, we highlight the gaps in this knowledge and point to directions for further research that may enhance the efficacy of PDT in the treatment of glioma.

## 1. Introduction

Cancers, a large and heterogeneous group of malignancies, are becoming an increasingly important cause of premature mortality worldwide [1,2]. Although primary tumors of the central nervous system (CNS) account for only 2% of all primary cancers, they cause 7% of the cancer deaths in people under 70 years of age [3,4]. Gliomas account for 24% of all the primary brain and CNS tumors [4]. These tumors are diverse in terms of cellular origin, genetic profile, and morphology but together have one of the most dismal prognoses of all cancers [5,6]. However, there is no standard treatment for stage IV. All glioblastomas eventually progress or recur [7]. The current standard of therapy, which includes surgical intervention, radiotherapy, and chemotherapy, is therefore far from sufficient [8]. Work is still underway to discover a new effective form of glioma therapy [9,10]. One of them may be photodynamic therapy (PDT) [11]. It involves the topical or systemic application of a photosensitive compound, a photosensitizer (PS), which accumulates in the affected tissues. The photodynamic reaction begins with the absorption of light by the PS in the target tissue, which triggers a series of photochemical reactions that lead to the generation of ROS [12,13]. In photodynamic therapy, there are two pathways for the generation of the cytotoxic ROS products responsible for the destruction of cancer cells [14]. In type I, the irradiated PS that is in the ground state (3PS) absorbs energy and is converted to the singlet state (1PS*, * excited state). Through an inter-system transition (ISC), the excited state (1PS*) can relax into the manifold of the triplet state (3PS*) [15]. Thus, the PS in the triplet state (3PS*) can undergo electron transfer with oxygen. The ROS produced include compounds such as hydrogen peroxide, superoxide anions, and hydroxyl radicals. They cause specific cellular damage and contribute to radical reactions [16,17]. Type I PSs are less oxygen-dependent and more promising in terms of their high therapeutic efficacy in the hypoxic tumor microenvironment [18]. However, most of the PSs reported to date are based on a type II photochemical mechanism, and only a few strategies can realize type I ROS production [14]. In a type II process, the energy from the PS triplet (3PS*) is transferred to triplet oxygen (3O_2_), forming cytotoxic singlet oxygen (1O_2_). This type of oxygen specifically interacts with various elements of the cell, initiating cell death [19]. Both type I and type II processes can occur simultaneously, with one dominating depending on the type of photosensitizer and its concentration. Currently, this method has been approved for the treatment of head and neck cancer, esophageal cancer, pancreatic cancer, prostate cancer, and esophageal squamous cell carcinoma. In addition, it is widely used in dermatology to combat precancerous and cancerous lesions [20]. Research focusing on the effective use of PDT in glioma therapy is already underway with promising results [21]. In our work, we present the molecular changes in glioma after photodynamic therapy. Based on the available literature, we analyze and systematize the influence of various molecules, proteins, transporters, and transmitters on the efficacy of PDT and the effect of PDT on their expression. We highlight the selected current and experimental drugs that, by affecting the molecules described, interfere with glioma growth, attenuate growth, and potentially increase the cytotoxic effects of PDT. In addition, we highlight the gaps in the current knowledge and point out directions for future research that may contribute to the efficacy of PDT for glioma.

## 2. Molecules Implicated in Resistance to Photodynamic Therapy

### 2.1. Vascular Endothelial Growth Factor

The vascular endothelial growth factor (VEGF) is the most important mediator of angiogenesis in glioma [22]. The formation of abnormal tumor vascularization is one of the main factors responsible for the resistance of these tumors to treatment, and the VEGF levels in glioma patients are higher than in healthy individuals [23,24]. Therefore, an increase in VEGF is particularly undesirable [24]. The ability of PDT to induce VEGF expression in both tumors and healthy brain tissue in a dose-dependent manner of the light used is well documented in the literature, and the mechanism of this phenomenon is due to the fact that, through hypoxia, PDT induces the stabilization of (hypoxia-inducible factor 1 α) HIF-1α [25,26,27,28,29,30,31]. Thus, stabilized HIF-1α accumulates in the nucleus and binds to HIF-1β, inducing the expression of a large number of proangiogenic factors, including the VEGF and VEGF receptors (VEGFR) [32]. A key approach to improving the efficacy of glioma PDT may therefore be to inhibit VEGF expression or block the adverse effects of its receptor. Combined PDT treatment with PS chlorin e6 (Ce6) and the humanized anti-VEGF monoclonal antibody bevacizumab prolonged the mean survival time of mice relative to PDT alone [33]. The association of PDT with PS photofrin (Ph-PDT) with anti-VEGFR-1 and anti-VEGFR-2 monoclonal antibodies reduced tumorigenesis more and prolonged mouse survival relative to PDT alone. Moreover, it also led to a significant reduction in the expression of both the VEGF and von Willebrand Factor [34]. The association of PDT with endostatin, an endogenous VGFR-inhibiting angiogenesis inhibitor, seems to be an interesting approach. It was previously observed that PDT with PS Hypocrellin-A and -B induces VEGF and endostatin release in glioma cells while reducing endostatin release in endothelial cells, and it has been shown that the predominance of the VEGF over endostatin observed early after PDT may contribute to enhanced angiogenesis [26,35]. This approach was tested by Zhan et al. By associating endostatin with HMME-PDT, they enhanced the effects of PDT, leading to greater tumor shrinkage, prolonged mouse survival, and decreased expression of HIF-1α and VEGF-A [36,37]. Important to the efficacy of VEGF-blocking treatment and PDT is the timing of the administration of the inhibitor as it has been shown that, when administered before PDT, it can reduce the PS levels and blunt the effects of the PDT [38]. However, it should be noted that the results of the clinical trials of antiangiogenic drugs in patients with malignant gliomas have been generally disappointing. Thus, a combination therapy with bevacizumab appears to be a particularly promising direction for future research as its use has previously been shown to benefit patients with recurrent glioblastoma [39]. Another approach to reduce unwanted increases in the VEGF may be to address hypoxia in PDT. For precise and accurate information on these, we recommend the paper by Larue et al. [40].

### 2.2. Hypoxia-Inducible Factor 1α

HIF-1α is a transcription factor that activates multiple glioma survival signaling pathways [41,42]. Its level is increased by an oxygen concentration of about 2.5% to 10%, found in most glioblastoma multiforme types [43]. Moreover, hypoxic tumors are three times more resistant to PDT [44]. It has been observed that the HIF-1 α expression increases after PDT in many cancers [45,46,47]. In the case of glioma, it increases with the time since PDT application [36]. Moreover, sublethal PDT induces an increase in the HIF-1α expression in healthy brain tissue as well, stimulating the expression of the ADAM17–EGFR–PI3K–Akt pathway and increasing the invasion of subsequently implanted glioma cells [48,49]. HIF-1α may be responsible for PDT resistance in several types of cancer [50,51]. Thus, it can be speculated that there is a mechanism whereby glioma cells attenuate the PDT response through increased HIF-1α levels, and PDT further exacerbates this effect through the additional induction of this factor induced by oxygen consumption, possibly ultimately leading to tumor proliferation, metastasis, and invasion. The work on solving this problem is still ongoing [52]. There is a report indicating that selecting the optimal therapeutic regimen may not negatively affect the HIF-1α expression. Li et al. showed that PDT with PS hematoprophyrin monomethyl ether (HMME-PDT) incubated 2 h in the dark with glioma cells and irradiated for 60 s with a 0.75 J/cm^2^ laser led to a decrease in the level of HIF-1α [53]. Another solution is to use a molecule that does not induce an increase in the HIF-1α levels. This was achieved by Xu et al., but the active photodynamic phthalocyanine-based molecular beacon they developed increased the VEGF expression [25]. A third approach may be the association of PDT with agents that reduce HIF-1α expression. A liposome composed of the HIF-1α inhibitor vitexin and PS indocyanine green (ICG) showed potent inhibition of the tumor cells and suppression of their migration in a dose-dependent manner [54]. The association of acriflavin, an inhibitor of HIF-1α/HIF-1β dimerization, with PDT with PS 5-aminolevulinic acid (5-ALA-PDT) reduced the expression of HIF-1α, GLUT-1, GLUT-3, and HK2, improved the efficacy of the treatment of refractory GBM, and reduced tumor cell invasion and migration [55]. Molecules that do not directly target this transcription factor can also lead to HIF-1α downregulation. The association of PDT with endostar, a recombinant human endostatin, led to prolonged survival of U251 glioma mice, significantly reducing the expression of both VEGF-A and HIF-1α [36]. Since HIF-1α is induced by hypoxia, an entirely different strategy is to develop nanoparticles that reduce the problem of hypoxia, on which advanced work with favorable results is still underway [56,57,58,59]. There are many possibilities in this field. Many approaches have been tested in various cancers, such as the use of hyperbaric oxygen therapy, the introduction of external oxygen carriers such as perfluorocarbons and hemoglobin, in situ O_2_-generating catalysts such as photosynthetic bacteria and catalase, or O_2_ providers [60].

### 2.3. Adenosine Triphosphate (ATP)-Binding Cassette Efflux Transporter G2

**Figure 1:**
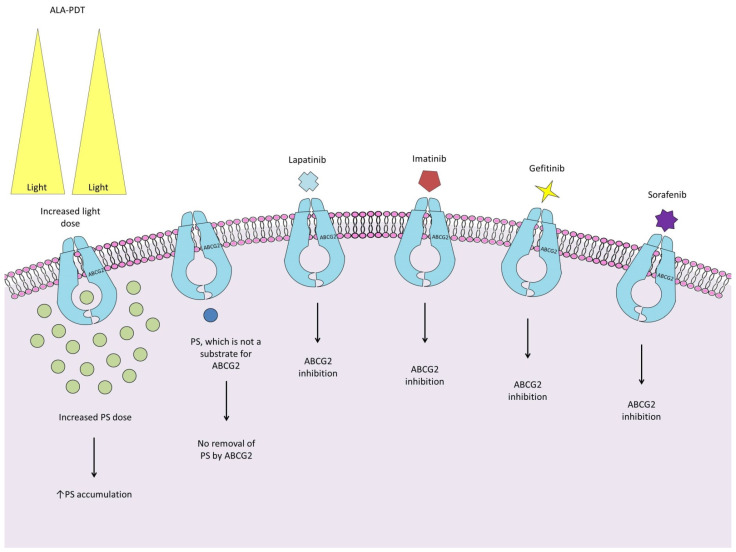
Strategies to improve the efficacy of photodynamic therapy for ABCG2-expressing glioma cells: (1) ABCG2 saturation and improved PDT efficacy can be achieved by increasing light and PS doses [62,63]; (2) increased PS concentration can be achieved by using strongly amphiphilic photosensitizers that are not substrates for ABCG2 [70]; (3), (4), (5), (6): association of PDT with ABCG2 inhibitors lapatinib, imatinib, gefitiniben, and sorafenib increases the efficacy of glioma PDT [62,68,71,72]. It should be noted that there are many more compounds already on the market waiting to be tested, such as telmisartan and febuxostat [75,76].

The adenosine triphosphate (ATP)-binding cassette efflux transporter G2 plays a central role in the efflux of various molecules from cells, providing protection against the adverse effects of xenobiotics [61]. Observations by Morgan et al. indicate that cells with ABCG2 overexpression are found only in a small group of U87 glioma side population (SP) cells, which also harbor resistant stem cells and may be a source of future tumor growth [62]. The involvement of ABCG2 in the response to PDT is not clear, and there are conflicting findings. Some reports indicate that glioma cells with high ABCG2 expression accumulate less photosensitizer, so ABCG2 may be responsible for the poor response to PDT [63,64]. This effect may be further enhanced by the PDT as hypoxia has been shown to increase ABCG2 expression [65]. In this work, the effects of PDT alone, and combined with the ABCG2 inhibitors KO143 and its analogue fumitremorgin C, at concentrations of 1.5 μM and 5 µM, respectively, were compared. However, Weidner et al. demonstrated that KO143 at concentrations ≥ 1 μM also affects the transport activity of both ABCB1 and ABCC1, which may have influenced the outcomes of these studies [66]. Wang et al. showed that the use of reserpine and verapamil, inhibitors of ABCG2, did not result in increased accumulation of protoporphyrin IX (PpIX) in both SP and non-SP cells. However, this may be due to their affinity for ABCB6, resulting in decreased synthesis of PpIX [67]. On the other hand, Abdel Gaber et al. restored the PS Ce6 accumulation using an anti-ABCG2 antibody, which would indicate a key role for this transporter in the removal of PSs [68]. The presence of ABCG2 alone does not indicate that glioma cells are resistant to PDT. The work of Hamid et al. achieved PDT efficacy despite 6-fold reduced PS photolon accumulation [69]. Regardless of the exact role of ABCG2, improving the phototoxicity of cells with the expression of this transporter is possible through several approaches. Some work indicates that ABCG2 saturation and improved PDT efficacy are provided by increasing light doses and PS [62,63]. Another of the strategies to solve this problem could be to use PSs that are not substrates for ABCG2, as demonstrated by Selbo et al. [70]. Another approach is to use ABCG2 inhibitors. Because of their ability to inhibit this transporter, clinically approved kinase inhibitors have been tested for this purpose. The results achieved vary depending on the inhibitor used. Further, 5-ALA-PDT in combination with imatinib was characterized by such high phototoxicity that the number of surviving SP cells was too small to measure [62]. Gefitinib, acting by reducing the mRNA and plasma membrane protein ABCG2 expression, increased the intracellular PpIX levels in a dose-dependent manner [71]. Lapatinib also increased the 5-ALA-PpIX fluorescence in a dose-dependent manner but was about 15% less effective than the KO143 inhibitor [72]. A similar effect was obtained using sorafenib, but, after its use, the Ce6 levels also failed to reach those achieved by KO143 or the anti-ABCG2 5D3 antibody [68]. However, kinase inhibitors do not selectively block ABCG2, so they may also increase the effectiveness of PDT by affecting other targets. It should be noted that, clinically, gefitinib was not very effective in treating GBM patients with EGFR amplification, while sorafenib promoted tumor cell proliferation in low-grade astrocytoma [73,74]. A number of ABCG2 regulators already introduced in the clinic, such as telmisartan and febuxostat, remain to be tested in glioma PDT association [75,76]. For precise and accurate information about them, we recommend the paper by Peña-Solórzano et al. [77]. Figure 1 shows the strategies to improve the efficacy of photodynamic therapy.

### 2.4. Nitric Oxide Synthase

Nitric oxide (NO), a free gas with numerous biological properties, at concentrations of 50–100 nM, can promote glioma growth, neovascularization, the immunosuppressive properties of the tumor microenvironment (TME), and resistance to therapies [78,79]. The gas also captures ROS, which can contribute to the overall resistance of cells to peroxidative stress and compromise the effectiveness of PDT treatment [80]. NO is mainly produced by the oxidation of L-arginine by three isoforms of NO synthase: inducible (iNOS), neuronal (nNOS), and endothelial (eNOS) [81,82,83]. While nNOS is the main synthase in the central nervous system, iNOS is most often associated with cancers, including glioma [81,83,84]. This synthase has been shown to function as a critical regulator of glioma development and is essential for brain tumor stem cell proliferation and tumorigenesis, and its increased expression has been described as a hallmark of chemoresistance in gliomas [84,85]. iNOS also has a negative impact on the efficacy of glioma PDT, increasing the resistance, survival, and migration of the surviving cells, including by playing a key role in the activation of Bcl-2 and survivin [86,87,88,89,90]. This effect is particularly important because it has been proven that, in an NF-kB-dependent manner, PDT can increase the iNOS levels in glioma cells by up to fourfold within 24 h of therapy [87,88,89]. However, the effect of PDT on nNOS levels has not been demonstrated [87]. An investigated proposal to solve this problem is the association of PDT with iNOS inhibitors. It was shown that the inhibition of iNOS activity closely matched the inhibition of glioma cell growth and invasion after PDT [88]. This approach is particularly attractive because, in numerous papers, iNOS inhibitors have shown significant potential as treatment options for oncologic lesions and have had a safe toxicity profile in humans for other pathological conditions [91]. On the other hand, higher NO concentrations of >300 nM have been shown to induce a cytotoxic effect on cancer cells, so a potential solution to the adverse effects of PDT on NO synthesis could be the development of a PDT therapeutic regimen characterized by significantly increased NO production [78]. There is still a lack of data on the relationship between glioma PDT and eNOS, and, given the importance of vascular effects in PDT efficacy, this issue requires clarification.

### 2.5. Glutathione

Acting as an antioxidant, glutathione (GSH) promotes tumor progression, and elevated levels correlate with temozolomide resistance and increased glioma metastasis [92,93]. High levels of intracellular GSH, which has the ability to scavenge free radicals, the primary mechanism of PDT efficacy, are also observed in PDT-resistant cells [94,95]. To address this issue and improve the efficacy of PDT, the blocker of reduced GSH synthesis, buthionine sulfoximine (BSO), was tested. Overnight treatment with 100 μM BSO followed by 4 h of coincubation with 5-ALA resulted in a nearly 100% reduction in the intracellular GSH of the T98G and U87 lines [96]. However, lowering the GSH did not always result in increased PDT efficacy. In the above study, a significant improvement in the ALA-PDT efficacy was achieved only in the T98G line, reducing the cell survival from 70 to 18% [96]. On the other hand, Jiang et al. reported that BSO already at the lowest dose used (0.225 μM) significantly increased the Ph-PDT activity in the U87 and U251n lines in vitro after 24 h coincubation, and this effect was dose-dependent. BSO also potentiated the effect of Ph-PDT in U87 cells in vivo [97]. Thus, the data are disparate, and the different PSs used, BSO dose, incubation time, and the fact that BSO itself exhibits cytotoxic activity make it difficult to interpret these differences and point to the need for further research [98]. However, the presence of GSH in glioma cells can also be used to improve the effect of PDT. Hwang et al. engineered PS ubiquinone-BODIPY, whose reduction by intracellular glutathione results in the increased generation of singlet oxygen [99]. GSH also enables PS and chemotherapeutic agent release, which is being taken into account in the design of new nanoparticles [100,101,102].

### 2.6. Ferrochelatase

Ferrochelatase (FECH) is an enzyme that converts photochemically active PpIX into photochemically inactive heme by incorporating ferrous iron [103]. The high expression and activity of this enzyme have been linked to glioma cell resistance to 5-ALA-PDT [96,104]. Moreover, hypoxia was found to cause increased ferrochelatase gene expression in glioblastoma stem cells, which may further contribute to 5-ALA-PDT resistance [105]. Therefore, iron chelation represents an attractive target that can enhance the efficacy of 5-ALA-PDT. Reburn et al. synthesized an ester between ALA and the hydroxypyridinone iron chelating agent CP94 and observed significant enhanced effects on both the PpIX accumulation and PDT cytotoxicity in glioma cells [106]. Also, the coincubation of CP94 with 5-ALA, methyl aminolevulinate (MAL), and hexyl aminolevulinate (HAL) increased the PpIX accumulation. Blake et al. compared these results with coincubation with dexrazosan, a clinically approved iron chelating prodrug, and found that it had less efficacy than CP94 [107,108]. Another approved compound tested for this purpose is deferoxamine (DFO), but the data on its efficacy are conflicting. A paper by Wang et al. showed that it effectively increased the PpIX accumulation in glioma lateral population stem cells [67]. On the other hand, Mansi et al. found that the addition of DFO led to only a slight increase in the PpIX fluorescence in all the glioma cell lines, significantly reducing the viability of only A172 cells [72]. It is difficult to determine the reason for this difference. The incubation time was identical in both studies at 4 h. A lower concentration of DFO (100 µM) was used in the first study than in the second (1 mM), yet DFO was found to be effective. Different cell lines were used in both works. This may have played a role in the discrepancy as Teng et al. showed that the ferrochelatase levels were the lowest in the U87 line and therefore did not use them to try to silence the enzyme [109]. However, this was the only common line also used by Mansi et al. In their work, the FECH activity measured in U87 cells was lower than in H4 cells, but, despite this, no significant differences in fluorescence were observed after the DFO treatment [72]. On the other hand, Blake et al. used the U-87 line and, as mentioned above, successfully inhibited FECH, but using compounds other than DFO [107]. The role of DFO in inhibiting glioma cell ferrochelatase is therefore unclear and requires further clarification.

### 2.7. Heme Oxygenase 1

Heme oxygenase 1 (HO-1) is an enzyme that catalyzes the oxidation of the heme group to form biliverdin. It also generates carbon monoxide and ferrous iron as reaction products. HO-1 and its reaction products have protective effects against oxidative stress, anti-inflammatory, and signaling effects [110]. The observations by Wang et al. indicate that the cells of the lateral population of glioma show significantly higher expression of HO-1. Moreover, the expression of this enzyme is much higher in WHO grade IV GBM. Higher levels of HO-1 expression may accelerate the PpIX/hem metabolic pathway, leading to poor 5-ALA-mediated PpIX accumulation and a low response to 5-ALA-PDT [67]. HO-1 has also been shown to be involved in PDT resistance in several types of cancer [111]. The expression of heme oxygenase 1 increases significantly after both ALA administration alone and subsequent irradiation [67,96]. The reduction in HO-1 activity may therefore contribute to the efficacy of the glioma therapy. Mastrangelopoulou et al. observed, however, that the synergistic effect of 5-ALA PDT and the HO-1 inhibitor OB24 was observed only in T98G cells preincubated overnight. They did not find it in other cell lines, nor during hourly preincubation [96]. Since HO-1 inducibility can also occur in response to the activation of growth factor receptor signal transduction pathways, including extracellular signal-regulated kinases ERK1 and ERK2, c-jun-NH2 kinase, and p38 kinase, in order to further reduce the negative effects of HO-1 on the efficacy of glioma 5-ALA-PDT, it seems attractive to test the molecules that affect these pathways [112,113,114].

### 2.8. Na^+^/H^+^ Exchanger Isoform 1

Na/H exchanger isoform 1 (NHE1) is a membrane Na^+^/H^+^ exchanger. It is one of the molecules that maintain the alkaline intracellular pH of glioma cells and the acidic TME [115,116]. NHE-1 is highly expressed in both glioma cells and tumor-associated microglia cells and macrophages [116,117]. Its activity increases the proliferation, invasion, and migration of glioma cells and contributes to TME immunosuppression and treatment resistance [116,117,118]. The relationship between PDT, NHE1, and glioma has been insufficiently studied to date. It has been established that NHE1 can attenuate the PDT effect of glioma through the increased extrusion of H^+^ [119]. It has been shown that 5-ALA-PDT and HMME-PDT can reduce the NHE-1 levels, and this effect is enhanced by associating them with dihydroartemisinin and temozolomide, respectively [119,120]. However, it should be noted that HIF-1α-dependent NHE1 expression is induced by hypoxia, so this issue needs further clarification [121]. An interesting as yet unexplored approach appears to be the association of PDT with the NHE1 inhibitor 5-benzylglycinyl-amiloride, which has shown anti-tumor activity in glioma [122]. It should be noted that NHE1 is not the only molecule that maintains the alkaline intracellular pH of glioma cells. It has been found that in gliomas these functions can also be performed by hypoxia-induced carbonic anhydrases (CA), particularly CAIX and CAXII [123,124]. Work is currently underway to use already marketed CA inhibitors, such as acetazolamide, for the treatment of glioma, and several have shown promising results in preclinical studies of brain tumors [123].

### 2.9. Other Molecules Involved in Glioma Resistance to PDT

Lee et al. indicated that a tumor protein (TP53) is involved in mediating Ph-PDT resistance by binding to the ALKBH2 promoter [125]. Park et al. suggested that the key molecule responsible for glioma PDT resistance is C5α [126]. Higher APE1 endonuclease activity and increased expression and activation of the DNA damage kinase ATM may also serve these functions [127].

## 3. Molecular Determinants for Photodynamic Therapy Resistance and Improved Photosensitizer Delivery in Glioma

### 3.1. Molecules without a Clear Role in Glioma Resistance Whose Regulation Enhances the Effect of PDT in a PDT-Dependent Manner

#### 3.1.1. Histone Deacetylases

Histone deacetylases (HDACs) are a group of enzymes that catalyze the removal of the acetyl groups from an ε-N-acetyl lysine amino acid on both histone and non-histone proteins [128]. There are several different classes of HDACs, the expression of which, depending on the class, is increased or decreased in the glioma [129]. PDT affects the expression and activity of HDACs. Single reports show an increase in the HDAC activity in A375 melanoma cells and healthy brain tissue [130,131]. Work is already underway to develop optimal inhibitors of HDACs; however, clinical trials have shown that they are currently ineffective in the treatment of GBM [132]. The HDAC inhibitor vorinostat in combination with the proteasome inhibitor bortezomib proved to be clinically ineffective in the treatment of recurrent GBM [133]. One of the inhibitors being tested is sodium butyrate (NaB). It has been shown that it can induce apoptosis, inhibit VEGF expression, and stimulate medulloblastoma differentiation [134,135,136]. The association of sodium butyrate with 5-ALA-PDT increased the U373-MG and D54-MG astrocytoma cell deaths by 67.8% and 53.95%, respectively, relative to 5-ALA-PDT alone [137]. Modified chromatin and NaB-induced genes are responsible for this effect [138]. A different approach was used by Wei et al. using HDAC6 to selectively activate their engineered molecule and enhance the effect of PDT [139]. In conclusion, the lack of knowledge about the HDACs and PDT in glioma is considerable, but the available work points to the potential use of these molecules and their inhibitors to improve the efficacy of glioma PDT.

#### 3.1.2. Nuclear Factor kB

Nuclear factor kB (NF-kB) comprises a family of transcription factors involved in the regulation of a wide variety of biological responses. NF-kB plays a well-known function in the regulation of the immune response and inflammation, as well as regulating the expression of the genes involved in many key processes of oncogenesis [140]. One of the cancers whose NF-kB hyperactivation can promote development, progression, and resistance to therapy is glioma [141,142]. NF-kB may have both positive and negative effects on the efficacy of glioma PDT; however, this issue is not completely clarified. NF-kB has been shown to have pro-apoptotic and anti-necrotic effects after PDT [143]. However, earlier work suggested both anti-apoptotic and anti-necrotic effects of NF-kB and a contribution to PDT resistance [144]. Thus, this issue requires further clarification. Moreover, PDT-induced NF-kB can also increase the migration of glioma cells [86]. On the other hand, NF-kB activation is important for the activation of the anti-tumor immune response by PDT [144]. PDT is not indifferent to NF-kB expression, either directly in the glioma or in other tissues. It has been shown that 5-ALA-PDT can lead to both the downregulation of NF-kB and enhancing its activation in glioma cells [143,145,146]. Moreover, low-dose PDT with PS Porfimer sodium induces the NF-kB of the TNF-α/NF-kB pathway in cerebral vascular endothelial cells and impairment of their function [147]. This effect may be important for the effectiveness of delivering other therapeutics to the tumor after PDT. Arguably, then, the goal of PDT in terms of its effect on NF-kB should be to modify the therapy in such a way as to achieve an optimal, not overly high or low, level of NF-kB expression. To achieve this, it is possible to test strategies based on both the selection of an appropriate PDT therapeutic regimen and the association of the PDT with NF-kB inhibitors. Coupienne et al. showed that the association of 5-ALA-PDT with an NF-kB inhibitor improves glioma cell death [143]. Work on the development and use of an effective NF-kB inhibitor in gliomas is still ongoing, creating the potential to test new combinations [148,149].

#### 3.1.3. Fibroblast Growth Factors

Fibroblast growth factors (FGFs) are mitogens that regulate a wide range of cellular functions, including migration, proliferation, differentiation, and survival [150]. Their receptors (FGFR) are overexpressed and mutated in many types of cancer [151]. High levels of FGF1 and FGF2 in glioma tissue contribute to angiogenesis, growth, invasion, and resistance to treatment of this cancer [151,152]. The reports on the effect of PDT on the FGF levels are conflicting and include both a decrease in its levels and an increase [153,154]. There are isolated papers describing the relationship between glioma, FGF, and PDT efficacy. Vilchez et al. showed that glioma cells resistant to PDT with methyl-5-aminolevulinic acid PS expressed higher mRNA levels of the fibroblastic growth factor receptor (FGFR), but this was not the only receptor upregulated [155]. However, it does not appear that FGF2 alone is crucial to the efficacy of 5-ALA-PDT as FGF2 added to U-105MG glioma cells did not have a stimulating or inhibitory effect on the final treatment outcomes [156]. However, this was an in vitro study, and, since FGF2 induces angiogenesis by activating endothelial cell proliferation and migration, the exact effect would need to be studied in vivo [151]. A new strategy leading to, among other things, the downregulation of the fibroblast growth factor receptor and increased apoptosis was developed by Chakrabarti et al. by associating Ph-PDT with the transfection of miR-99a [157]. Some of the selective FGF/FGFR inhibitors have already been approved for therapy, and testing them in combination with PDT could be an interesting direction to potentially increase the efficacy of PDT and help to further clarify the relationship between PDT, FGF/FGFR, and glioma [158]. However, it should be noted that, clinically, they did not achieve success. Those patients treated with the Pan-FGFR kinase inhibitor Erdafitinib had only a partial response, and those treated with the FGFR and VEGFR inhibitor dovitinib had no success [159,160].

#### 3.1.4. Glutamate

Glutamate (GLU) is an excitatory neurotransmitter (NS) that plays a central role in glioma malignancy [161,162]. It is produced and released from cells as a by-product of glutathione synthesis [161]. PDT can increase the GLU levels. HpD-PDT has been shown to induce a sharp threefold increase in the glutamate levels that persisted for at least 16 h [163]. GLU induces glioma malignancy through multiple mechanisms, including excitotoxicity to the surrounding healthy neurons and paracrine and autocrine effects on the glutamine receptors [161,162,164]. It was shown that HpD-PDT increased the GluR1 and GluR2 AMPAR subunit expression, Ca2+ influx, and apoptosis of C6 glioma cells [163,165]. This effect may be exacerbated by the PDT-induced release of glutamate [163]. However, it should be emphasized that the aforementioned work was conducted in vitro and therefore could not verify the adverse effects of the PDT-increased glutamate concentrations on the healthy brain tissue surrounding the tumor. This issue requires necessary clarification. Data on the effects of PDT on NMDA receptors are also lacking. The GLU inhibitors tested in glioma could presumably be useful in regulating the effects of PDT on glutamate receptors [161]. The combination of PDT with a metabotropic glutamate receptor 5 inhibitor seems particularly attractive as it has been shown to be effective in facilitating hypoxia-induced glioma cell death [166].

#### 3.1.5. Peripheral-Type Benzodiazepine Receptors

The peripheral-type benzodiazepine receptor (PBR) is a mitochondrial protein that is expressed at high levels in steroid-synthesizing tissues, including brain glial cells [167]. Basal PBR expression is upregulated in gliomas [168,169]. It has been suggested that the relatively high binding density of peripheral-type benzodiazepine receptors is associated with increased tumorigenicity and rates of cell proliferation [170,171]. It is known that PDT is not indifferent to PBR expression in glioma. Bisland et al. showed that ALA-PDT with low light levels increased the PBR expression in a CNS-1 glioma model [172]. The regulation of PBR expression by PDT has implications for the efficacy of therapy, but there are few reports in this area. A study on mouse leukemia cells showed that the affinity of PpIX-configured PS for PBR can affect the efficacy of PDT [173]. Further, the 5-ALA-PDT-induced increase in PBR facilitated increased production of PpIX and enhanced phototoxic effects against glioma cells [172]. The potential beneficial effect of the presence of PBR on the efficacy of glioma PDT was exploited by Sarissky et al. They tested the association of Hy-PDT with diazepam, a non-selective PBR ligand with established activity to inhibit glioma cell proliferation [174,175]. They showed that the combination enhanced cell apoptosis, but only of the U-87 line, with increased PBR expression relative to the U373MG line, which was resistant to the combination. However, they did not describe the effect of Hy-PDT on the PBR expression levels [174]. Significantly, the association of diazepam with the chemotherapeutics tested to date has resulted in both improved efficacy and reduced efficacy, and glioma cells with high nuclear PBRs proliferate in response to PBR ligands [171,176,177]. Thus, further work is needed to precisely describe the relationships between PDT, the PS used and the light dose applied, PBR expression, and the ultimate impact on the effectiveness of the glioma therapy. Both agonists and antagonists for these receptors are already proven and available in the clinic.

#### 3.1.6. Matrix Metalloproteinases

Matrix metalloproteinases (MMPs) are a group of enzymes that catalyze the remodeling of the extracellular matrix [178]. They play a key role in the mechanisms of glioma invasion, and the increased expression of some of them has been linked to poor prognosis and glioma recurrence [179,180]. PDT leads to decreased expression of the glioma MMPs (-2, -7, -8, and -9) and cell migration, which is well documented in the literature and consistent with the observations from other cancers [181,182,183,184,185,186,187]. It has been shown that the association of MMP inhibitors with PDT can potentially enhance the clinical effect of PDT, so an interesting direction for future work seems to be the association of PDT with the MMP inhibitors tested in glioma [188,189].

#### 3.1.7. PD-L1/PD-1

The axis of programmed cell death protein 1 (PD-1) and programmed cell death ligand 1 (PD-L1) plays an important role in inhibiting the host immune response against cancer cells [190]. Studies have shown that the PD-1/PD-L1 pathway plays a key role in glioma progression and the effectiveness of immunotherapy [191]. There is a dearth of work to date describing the effects of PDT on the PD-1/PD-L1 expression in tumors, and the available results indicating that PDT can both block and enhance the PD-1/PD-L1 axis activity are contradictory [192,193,194]. However, none of the papers cited used glioma cells for the study. Recently, the ability of PDT to induce immunogenic cell death (ICD), activate a tumor-specific immune response, and induce a long-lasting and effective anti-tumor response has been of great interest [195,196,197]. Turubanova in et al. showed that repetitive PDT with PS photosens and photoditazine can achieve this goal [198]. Shibata et al. also induced the ICD of glioma cells via liposomally formulated IG conjugated to phospholipids [199]. ICD is also desirable for glioma therapy and can improve the results of immune checkpoint blocker therapy [200,201]. The utility of the potential use of PDT for this purpose is confirmed by the work of Xu et al. They showed that the association of PS Ce6 with αPD-L1 resulted in greater infiltration of immune effector cells and prolonged life in mice with orthotopic GL261 glioma compared to PDT or αPD-L1 alone [202]. PDT-induced ICD and the downregulation of PD-1 and PD-L1 by the deprivation of extracellular cholesterol in glioma TME also induced nanoparticles (NPs) designed by Yin et al. [203]. The combination of the PD-1/PD-L1 axis blocking method, PDT, and dendritic cell (DC) therapy seems to be an interesting approach. The presence of PD-1 may adversely affect the efficacy of dendritic cells, which dysfunctionally limit the antigen-specific T-cell responses in glioma [204,205]. Several works have shown that glioma PDT with different PS can improve the DC functionality [206,207,208,209]. Since the anti-PD-1 treatment during DC maturation resulted in increased DC survival, the approach of combining PDT and a method that blocks the PD-1/PD-L1 axis could therefore be an attractive improvement on the hitherto suboptimal results of glioma dendritic cell therapy [204,210].

#### 3.1.8. Transporter Associated with Antigen Processing 1

Transporter associated with antigen processing 1 (TAP1) is a molecule involved in the processing and presentation of antigens restricted to the major class I tissue compatibility complex, including tumor-associated antigens [211]. TAP1 is involved in tumor immunity and is abnormally expressed in many types of cancer, including glioma, which may be less immunogenic due to low levels of TAP1 expression [212,213,214]. Since it has been shown that high TAP1 expression resulted in better patient responses to anti-PD-1, anti-PD-L1, and anti-CTLA-4 immunotherapy in several cancers, increasing the TAP1 expression presumably could be an attractive form of improving the hitherto unsatisfactory results of the immune checkpoint blockade in glioma therapy [211,215]. The work of Zhang et al. showed that PDT may have this ability. They showed that HMME-PDT enables glioma cells to recover both the expression of functional TAP1 and the presentation of MHC class I surface antigens [216]. However, this is the only report in the field so far, so it needs to be confirmed in other works.

#### 3.1.9. GLUT Glucose Transporters

Two transporters are involved in the glucose transport into cells. The first, GLUT, mediates facilitated passive transport, while the second, SGLT, mediates active transport. The expression of GLUT and SGLT is tissue-dependent and depends on its role in carbohydrate metabolism [217,218]. The complex role of GLUTs in glioma has not yet been completely elucidated. Specific functions are known to vary for different types of GLUTs, but it has been established that GLUTs may be responsible for the proliferation and invasion of the tumor [219,220,221]. Liu et al. found that the GLUT-3 expression in glioma was significantly correlated with the pathological grade of the tumor [219]. The effect of PDT on the GLUT levels in glioma is unclear, and the available data are conflicting. Li et al. reported that HMME-PDT inhibits the expression of GLUT-1 [53]. On the other hand, Ma et al. showed that the expression of GLUT-1 and GLUT-3 was significantly increased after 5-ALA-PDT [55]. This effect is presumably dependent on HIF-1*α*. In the first study, PDT led to a decrease in it, while, in the second, an increase was observed. This is consistent with previous findings on the effect of HIF-1*α* on GLUT-1 expression [220]. In view of this, the regulation of HIF-1*α* expression may be an attractive way to reduce GLUT expression. Because of their effect on GLUT tumor growth, they can be used to improve the effects of PDT. Zhang et al. achieved this by synthesizing a novel PS that effectively targeted the GLUT/hexokinase pathway [222]. Moreover, by modifying drugs with glucose, GLUTs can also be used to achieve improved crossing of biological barriers and targeted delivery of therapeutics [223]. This property has also been successfully exploited in association with PS, but not in glioma therapy, where the association of PS with other sugars has been tested [224,225].

### 3.2. Molecules without a Clear Role in Glioma Resistance Whose Regulation Enhances the Effect of PDT in a PDT-Independent Manner

#### 3.2.1. Protein Kinase C

Histone protein kinase C (PKC) is a family of serine/threonine-specific protein kinases [226]. The PKC activity was found to be increased in gliomas compared to astrocytes [227,228]. PKCs play a diverse role in glioma development as the contribution of each PKC isoform depends on the phosphorylation of tyrosine residues, the presence of oncogenic mutations, the type of stimuli, and the cellular environment [226]. However, it has been shown that PKCs can contribute to tumor resistance to PDT [229]. Previous work has shown that PDT affects the PKC activity of glioma cells, both through the action of PS alone and subsequent irradiation. Huntusova et al. reported that hypericin interacts directly with PKCα and increases the PKCδ autophosphorylation in glioma [230]. This issue requires clarification as there are reports indicating that hypericin can inhibit the PKC in other cells [231]. The data on the effects of PDT on PKC in glioma are limited. Uzdensky et al. demonstrated that sublethal 5-ALA-PDT induces an increase in PKCγ and PKCβ [232]. The effect of PDT on PKC is confirmed by the work of Dzurov et al., who showed that Hypericin-PDT (Hyp-PDT) caused the activation of PKCα [233]. On the other hand, Pevna et al. achieved a decrease in the PKCα levels by Hyp-PDT [234]. Since both Dzurova et al. and Pevna et al. used U87, Hyp cells at a concentration of 500 nM, but different light doses (4 J/cm^2^ and 2 J/cm^2^, respectively), the light dose may be crucial for the PKCα regulation in glioma. Given the involvement of PKCα and PKCβ in the survival, proliferation, and migration of glioma cells, the potential possibility of their regulation by an appropriately selected light dose seems particularly attractive [226]. An approach being tested to improve the efficacy of glioma PDT is association with PKC inhibitors. Tamoxifen at a dose of 500 μg/mL and higher was shown to significantly increase the toxicity of the Ph-PDT response [235]. The work on using tamoxifen for this purpose is particularly promising as it has shown potential in treating glioma [236,237]. The pretreatment of glioma cells with rottlerin before Hyp-PDT led to a significant increase in apoptosis [238]. However, recent work has challenged the previous findings regarding the main function of rottlerin as a PKCδ inhibitor [239,240,241]. A completely different approach is the association of photobiomodulation with PDT. It was shown that photobiomodulation performed before Hyp-PDT with 500 nM Hyp led to a greater decrease in PKCα than Hyp-PDT alone [234]. In conclusion, PKC targeting may be an attractive form of improving the effectiveness of PDT, but there is still much to be clarified.

#### 3.2.2. Hepatocyte Growth Factor

Hepatocyte growth factor (HGF), also called scatter factor, along with its receptor tyrosine kinase c-Met, are key determinants of brain tumor growth and angiogenesis [242]. HGF can affect tumor progression through various mechanisms, including modulating the cell growth, migration, and chemoresistance to drugs [243]. There is one paper describing the effect of PDT on HGF. The study by Vogel et al. showed that 5-ALA-PDT induced HGF expression in glioblastoma spheroids and thus stimulated the migration of mesenchymal stem cells into the tumor. Moreover, they concluded that both the increases in HGF expression and MSC migration after PDT can have positive and negative effects on the ultimate efficacy of the therapy [244]. The development and effective use of the HGF inhibitor are already underway [245]. It should be noted, however, that their clinical testing to date has not shown promising results [9].

#### 3.2.3. Vascular Cell Adhesion Protein 1

Vascular cell adhesion protein 1 (VCAM-1, CD106) is a glycoprotein involved in angiogenesis and cancer metastasis [246]. VCAM-1 is highly expressed in both gliomas and the surrounding normal brain tissue [247,248]. It has been shown that PDT can induce an increase in the VCAM-1 levels in glioma [249]. However, the available data on the effects of PDT on the VCAM-1 expression in tumors are conflicting, so this issue requires additional clarification [250,251]. Due to its functions and the high expression of VCAM-1 in glioma, targeting this molecule may be a strategy to increase the effectiveness of the therapy. Zhan et al. showed that the combination of HMME-PDT with a monoclonal antibody against VCAM-1 induced apoptosis and prolonged the mouse survival more than PDT alone [249].

#### 3.2.4. Glial Fibrillary Acidic Protein

Glial fibrillary acidic protein (GFAP) is an intermediate filament protein occurring in several isoforms, characteristic of astrocytes and neural stem cells and their malignant analogs in glioma [252,253]. The role of GFAP in the development of glioma aggressiveness is complex. Cells with a high GFAPδ/α ratio can be highly malignant and more invasive [254]. Moreover, the deprivation of any of these isoforms has also been shown to increase the migratory capacity of glioma cells [255]. The effect of PDT on GFAP expression has not yet been precisely established. Namatame et al. reported that PDT with PS talaporfin sodium decreased GFAP expression, leading to the disappearance of GFAP expression [256]. However, it is difficult to assess the effect of GFAP knockdown on the efficacy of PDT. On the one hand, this effect in the long term may be unfavorable as GFAP-negative cells have been shown to have a higher average number of nucleolar organizer regions (Ag-NOR), an increase in which has been linked in a couple of papers to a worse prognosis in several cancers, including gliomas [257,258,259,260]. On the other hand, however, there are no reports on the effect of PDT on Ag-NOR, so it is difficult to infer whether a decrease in GFAP by PDT can lead to an increase in Ag-NOR. For obvious reasons, this inference also does not take into account the percentage of GFAP-negative cell death after PDT. Namatame et al. also did not determine the dynamics of the changes between the GFAP isoforms, which may be crucial in determining the benefit of PDT in this regard [256].

#### 3.2.5. Receptor-Interacting Serine/Threonine Kinases

Receptor-interacting protein kinases 1 (RIP1) and 3 (RIP3) are regulators of programmed necrosis [261]. The exact function of the RIP1/RIP3 pathway in cancer development has not been elucidated, and both the stimulation and inhibition of this pathway have been proposed for anti-cancer therapy [262]. Several of the compounds tested in glioma therapy have been shown to induce the RIP1/RIP3 pathway to induce the necroptosis of its cells [263,264,265]. The RIP1/RIP3 complex regulates programmed glioma necrosis even after a high dose of radiation [266]. The effect of PDT on RIP1/RIP3 is not established. Coupienne et al. showed that the 5-ALA-PDT of glioblastoma cells activates the RIP3-dependent necrotic pathway, and the resulting necrosome is composed of RIP1, RIP3, and other unrecognized stubs [267]. Fettweis et al. characterized the RIP3/TSC2 complex, suggesting that RIP3 promotes glioma cell death by targeting the TSC2-dependent survival pathway after 5-ALA-PDT [268]. Since work on the drugs affecting the RIP1/RIP3 pathway is already underway, a thorough elucidation of its role in glioma PDT is essential for their successful association [262].

### 3.3. Other Molecules Affecting the Efficacy of Glioma PDT with No Established Role in Resistance

Other works present further molecules that affect the effectiveness of PDT. The organic arsenic molecule PDT-BIPA created by Liu et al. remodeled the metabolic pathway by targeting lactate dehydrogenase A (LDHA). The PDT-BIPA precursor directly inhibited the LDHA function and converted glycolysis to oxidative phosphorylation, causing an explosion of ROS and mitochondrial dysfunction. PDT-BIPA also led to the altered expression of several genes, such as HIF-1α and C-myc [269]. PDT-BIPA also led to the upregulation of thioredoxin, which is a major antioxidant system integral to maintaining the intracellular redox state [269,270]. Wu et al. reported that the presence of intercellular communication through Connexin Cx43 improves the phototoxicity of glioma PDT [271]. An et al. demonstrated that PDT with PS Sinoporphyrin sodium reduces the protein phosphorylation of the PI3K/AKT/mTOR signaling pathway [272]. The effect of PDT on the immune response is highlighted by Li et al., demonstrating that glioma PDT also induces the release of inflammatory factors such as TNF-α and IFN-γ [273].

## 4. Molecules Used to Improve Photosensitizer Delivery

### 4.1. Epidermal Growth Factor Receptor

**Figure 2:**
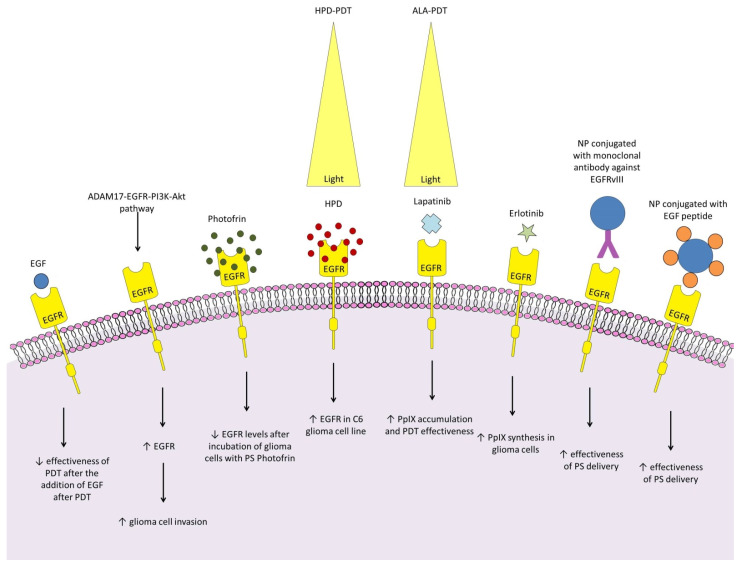
(1) Relationships between photodynamic therapy, glioma, and EGFR: EGF had no effect on PS hematoporphyrin derivative (HPD)- and laser-induced toxicity when added to cells before PDT, but added after attenuated the cellular response to PDT [278]; (2) Ph-PDT can induce the ADAM17–EGFR–PI3K–Akt pathway in healthy brain tissue, leading to an increase in EGFR in glioma cells as well as healthy brain cells, significantly increasing the invasion of U87 cells in nude mice [49]; (3) incubation of T98G cells with PS Photofrin led to a decrease in EGFR levels [279]; (4) HpD-PDT increased EGFR in C6 cells, but not T98G and U87 cells [235]; (5) the association of lapatinib with 5-ALA-PDT led to a significant increase in PpIX accumulation and induced stronger responses of two human glioma tumors in vivo, leading to increased survival in rats compared to lapatinib alone and PDT alone [283]; (6) erlotinib improved the selectivity of PpIX accumulation as it increased PpIX synthesis in glioma cell lines [282]; (7), (8) coupling NP with a monoclonal antibody against EGFR or EGF peptide improves the efficiency of PS delivery to the glioma [286,287].

The epidermal growth factor receptor (EGFR) is a transmembrane glycoprotein and belongs to the tyrosine kinase receptor superfamily [274]. High EGFR levels contribute to glioma development, progression, and resistance in both kinase-dependent and -independent ways [275,276]. The exact effect of the EGFR on the efficacy of glioma PDT has not yet been thoroughly investigated; however, the available work suggests that the EGFR may contribute to resistance to PDT, as it does in other cancers [277]. It has been shown that Ph-PDT can induce the ADAM17–EGFR–PI3K–Akt pathway in healthy brain tissue, leading to an increase in the EGFR in both healthy cells and subsequently implanted glioma cells, significantly increasing the U87 cell invasion in nude mice [49]. The effect of EGF, an EGFR ligand, is also unexplained. Fanuel Barret et al. showed that it had no effect on the PS hematoporphyrin derivative (HPD)-induced and laser-induced toxicity when added to cells before PDT, but, added after, it attenuated the cellular response to PDT. The data on the direct effect of PDT on the EGFR expression by glioma cells are inconclusive and disparate. Fanuel Barret et al. showed that incubation with PS HPD and subsequent laser irradiation had no effect on the increase in the EGFR in T98G and U87 cells but resulted in its increase in the C6 line [278]. On the other hand, in the work of Yang et al., the incubation of T98G cells with PS Photofrin led to a decrease in the EGFR levels [279]. It seems to be an interesting direction for future work to test the effect of ALA-PDT on the EGFR expression by glioma cells as testing other cancer cells has demonstrated the potential to lead to a reduction in the EGFR levels [280]. Given the important involvement of the EGFR in cancer pathogenesis, the attempts to combine PDT with EGFR-targeting molecules are currently attracting considerable interest [281]. For glioma, the focus was on combining PDT with clinically accepted EGFR inhibitors. Erlotinib improved the selectivity of PpIX accumulation as it increased the PpIX synthesis in the glioma cell lines while not affecting the PpIX synthesis in the neuronal and astrocytic lines [282]. The association of lapatinib with 5-ALA-PDT led to a significant increase in the PpIX accumulation in glioma cells and induced stronger responses of two human glioma tumors in vivo, leading to increased survival in rats compared with lapatinib alone and PDT alone [283]. A number of EGFR inhibitors remain to be tested in terms of glioma PDT association. For precise and accurate information about them, we recommend the paper by Abourehab et al. [284]. However, it should be emphasized that none of the EGFR-targeted therapies have shown promising results in clinical trials in patients with glioma, so these positive results of the work on the combination of PDT with EGFR inhibitors should be treated with caution and need to be confirmed clinically [285]. EGFR and EGF have also been successfully used as targets to improve the PS delivery to glioma cells [286,287]. Figure 2 shows the relationships between photodynamic therapy, glioma, and EGFR. 

### 4.2. Neuropilin-1

Neuropilin-1 (NRP-1) is a transmembrane protein involved in glioma proliferation, invasion, and migration, as well as tumor angiogenesis [288]. This molecule is overexpressed in the glioma [289,290]. NRP1 expression has been shown to be correlated with poor prognosis, glioma grade, and associated with the mesenchymal tumor subtype [291]. Previous work has shown that the association of PS with NRP-1-targeting molecules promotes the vascular effect of PDT, ultimately resulting in reduced tumor blood flow and delayed tumor growth [292,293,294,295,296,297]. This coupling has a higher efficiency than the PS itself [298]. Neuropilin-1 has also been successfully used as a target to improve the PS delivery to glioma cells [299].

### 4.3. Integrins

Integrins (INTs) are a large family of molecules and receptors on the cell surface, consisting of eighteen α subunits and eight β subunits, enabling the formation of twenty-four unique INTs [300]. They are important surface adhesion receptors that mediate the interactions between the extracellular matrix and cells and are essential for cell migration and the maintenance of tissue homeostasis. The abnormal activation of integrins promotes the initial formation, growth, and metastasis of tumors [301]. INTs are among the major contributors to the invasive glioma phenotype, particularly the abnormal expression of β1 subunit, α3β1 integrin, αvβ3, and αvβ5 integrin [302]. The beneficial effects of PDT on the integrin levels in tumors have already been fairly well studied [303]. A similar effect of PDT was demonstrated in glioma. The administration of the PS alone could reduce the level of integrin α3β1 in the glioma, and subsequent irradiation exacerbated this effect depending on the light dose, the PS applied, and the glioma cell line [181]. These observations are consistent with those of other cancers [303,304]. An interesting direction for future work seems to be the enhancement of the beneficial effect of PDT on integrin levels by association with the inhibitors of these molecules [305]. Cilengitide, an inhibitor that targets αvβ3 and αvβ5, has not shown clinically significant potential as a monotherapy; however, some patients have achieved benefits regarding its use as a combination treatment [9]. Integrins can also be used as useful targets to improve PS delivery and hit nanoparticles [299,306].

### 4.4. Neuropeptide Y Receptors

Neuropeptide Y receptors (NPYRs) are a group of several receptors (Y1R, Y2R, and Y5R) that mediate the action of the multifunctional neurotransmitter neuropeptide Y. They are characterized by proliferative effects, but their role has not been completely elucidated [307]. The NPYR expression in glioma varies according to the type of tumor. WHO grade IV gliomas are characterized by extremely high Y2R expression of the NPY receptor in both frequency and density; WHO grade I to III astrocytomas and oligodendrogliomas also show high Y2R frequency but low density, and medulloblastomas show Y1R and Y2R expression in moderate frequency and density [308]. The presence of NPYRs on glioma cells can be effectively used to target gliomas, as demonstrated in several papers [309,310]. This advantage can also be used to improve the PS delivery and PDT effects, as demonstrated by He et al. by equipping their designed NP with the Y1R ligand [311]. However, there is a lack of work on targeting Y2R, which could be particularly attractive in the treatment of WHO stage IV gliomas [308]. Since NPYRs are characterized by proliferative effects, it is also worth checking the effect of PDT on their expression levels and the relationship between expression and PDT efficacy. Neurotransmitters in glioma photodynamic therapy are shown in Table 1.

### 4.5. Low-Density Lipoprotein Receptor

The low-density lipoprotein receptor (LDLR) is highly expressed in blood–brain barrier and glioma cells, while normal brain tissues and neurons have relatively low levels of it [312,313]. Previous work has shown that targeting LDLRs may be a promising strategy for improving the drug delivery in glioma therapy [314]. This approach was also tested in PDT, associating LDLs with hydrophobic PSs [315,316,317,318]. The PDT-induced phototoxicity of glioma cells with berberine and hypericin PSs was correspondingly higher when combined with LDLs than when used alone [317,318]. However, the above results were described in vitro under conditions that do not test the ability to cross the BBB, so they would need to be confirmed in vivo. Since the efficacy of the PS–LDL association has been demonstrated in vivo in subcutaneously implanted HepG2 cells in nude mice, and targeting LDLR is a promising strategy for overcoming BBB limitations, this line of future research seems to be particularly warranted [314,319].

### 4.6. Other Molecules Used to Improve Photosensitizer Delivery

Several papers point to potential molecules that improve PS delivery. Akcker et al. showed that the association of PS with an antibody targeting the additional A domain of fibronectin leads to microvascular dysfunction and reduced glioma growth within the first 48 h after the treatment, with complete recovery 5 days after the treatment [320]. De Groof et al. indicate that improved PS selectivity can be achieved by coupling PS to NPs targeting a variety of G protein-coupled receptors [321]. This function can also be performed by nucleolin and the receptor for transferrin [322]. Akhlynina et al. showed that the nuclear targeting of photosensitizing conjugates by incorporating a large-tumor antigen nuclear localization signal results in enhanced photodynamic activity compared to free Ce6 [323]. Table 2 shows molecular targets tested to improve photosensitizer delivery to the glioma.

## 5. Conclusions

In this section, we present a summary of our work and the general conclusions. The analyses and conclusions on specific molecules can be found in the sections describing them. The recent advances in the fundamental studies of molecular PDT were presented here [324,325,326]. Despite the ongoing work, much is still left to be clarified. There is a lack of accurate knowledge about the effects of PDT on some molecules that are important for the effectiveness of glioma therapy, such as transforming growth factor β (TGF-β), and the information on some of them (e.g., HGF, HDACs, GFAP, and others) is far from sufficient. Because of the promising results of immune-based therapies, and the well-established ability of PDT to induce ICD, it seems particularly important to establish the precise relationship between PDT and the molecules affecting the effectiveness of immunotherapy, where there is still much to be clarified [327]. Clearly, further identification and investigation of the molecules contributing to glioma cell resistance to PDT are also needed. A thorough elucidation of the gaps in the current knowledge may prove crucial as the effects of PDT on specific molecules may vary depending on the therapeutic regimens and photosensitizers used, and it has been shown that the negative effects of some molecules (e.g., ABCG2) can be reduced or completely eliminated by using the appropriate therapeutic regimen or photosensitizer. However, the information in this area is far from sufficient. Because of the differences in the molecular function of different types of gliomas, it is important to tailor the molecular approach to a specific type of cancer [328]. There is also a lack of work describing the molecular basis of glioma PDT more broadly, taking into account the interactions between specific molecules. The previous work has mostly focused on the study of single molecules, which does not reflect the complex interactions between them. Moreover, the ultimate efficacy of PDT is influenced not only by the preservation of glioma cell molecules but also by the effect of PDT on the molecules of healthy brain tissue. In this regard, only isolated information is available and further work is needed. For this reason, the promising results of in vitro studies should also be tested in vivo under conditions that include the effects of PDT on healthy brain tissue. For the validity of the results of future studies, it is important to standardize the work on the analysis of the molecules involved in glioma PDT. As mentioned above, there are doubts about the selectivity of some of the inhibitors used in the studies, which may lead to discrepancies in the results and conclusions.

## Figures and Tables

**Table 1:** Neurotransmitters in glioma photodynamic therapy. In this table, we have collected available information on neurotransmitters described in photodynamic therapy of glioma. The second through fourth columns, respectively, locate data on the effect of PDT on the level of a neurotransmitter, the expression of its receptor, and the effect of a neurotransmitter on the efficacy of PDT. In the fifth column are the neurotransmitter-related approaches tested in PDT of gliomas that are related to the described neurotransmitters.

Neurotransmitter	NS Levels	Receptor Expression	Impact on the Effectiveness of PDT	Tested Therapeutic Approach
NO	Increase [87,88,89]	No data available	Increased resistance, survival, and migration of glioma cells after PDT [86,87,88,89]	Association of PDT with iNOS inhibitors reduces NO levels and glioma cell growth and invasion after PDT [88]
Glutamate	Increase [163]	Increase in AMPAR expression [163,165]	Increase in apoptosis of glioma cells [163,165]	No data available
GABA	No data available	Increase in PBR expression [172]	Increased production of PpIX and phototoxic effect against glioma cells (via increase in PBR) [172]	Association of PDT with diazepam enhances apoptosis of glioma cells [174]
Neuropeptide Y	No data available	No data available	No data available	NP coupling with NPY improves targeting and PS delivery in the glioma [311]

**Table 2:** Molecular targets tested to improve photosensitizer delivery to the glioma. The first column presents the molecular target used. In the second is the molecule responsible for the targeted delivery, which was conjugated to either a photosensitizer or a photosensitizer-loaded nanoparticle.

Molecular Target	Targeting Method	Article
EGFR	Monoclonal antibody against EGFRvIII	Jamali et al. [286]
EGFR	EGF peptide	Meyers, J.D. et al. [287]
Neuropilin-1	ATWLPPR heptapeptide targeting NRP-1	Thomas et al. [292] Tirand et al. [293] Bechet et al. [294] Tirand et al. [297] Thomas et al. [298]
Neuropilin-1	KDKPPR peptide targeting NRP-1	Gries et al. [295] Thomas et al. [296]
Neuropilin-1 Integrin αvβ3 Integrin αvβ5	RGD internalizing peptide targeting NRP1, INT αvβ3, and αvβ5	Lu et al. [299]
Integrin αvβ3	Modified monoclonal antibody against INT αvβ3	Wei et al. [306]
Neuropeptide Y receptor 1	Neuropeptide Y type D	He et al. [311]
Low-density lipoprotein receptor	Low-density lipoprotein	Andreazza et al. [317] Huntosova et al. [318]
Extra domain A of fibronectin (ED A)	Ligand targeting epitope ED A small immunoprotein antibody F8	Acker et al. [320]
US28 protein	Nanoprotein binding discontinuous epitope US28 with high affinity	De Groof et al. [321]
Nucleolin	Single-stranded DNA aptamer AS1411 with high affinity for nucleolin	Zhu et al. [322]
Transferrin receptor	Transferrin	Zhu et al. [322]
Cell nucleus	T-ag antigen variants of SV40 virus	Akhlynina et al. [323]
